# Pots syndrome: the importance of organic screening in anxiety patients. Case report

**DOI:** 10.1192/j.eurpsy.2022.1206

**Published:** 2022-09-01

**Authors:** P. Del Sol Calderón, L. Mallol Castaño, R. Paricio Del Castillo, A. Izquierdo De La Puente, M. Garcia Moreno

**Affiliations:** 1 Hospital Universitario Puerta de Hierro, Psiquiatría Infanto-juvenil, Madrid, Spain; 2 HOSPITAL UNIVERSITARIO PUERTA DE HIERRO MAJADAHONDA, Psychiatry, MADRID, Spain

**Keywords:** conversive syndrome, psychosomatic, POTS, functional symptoms

## Abstract

**Introduction:**

16-year-old female who starts psychiatric follow-up due to episodes of anxiety crises with dizziness and tremors.

**Objectives:**

To expose a case in which it is essential to rule out organic pathology in cases where there is anxiety and physical symptoms.

**Methods:**

Case report and literature review

**Results:**

The patient explains that she was in a situation of conflict with her ex-partner, commenting that he did not accept the breakup. Sertraline was prescribed in ascending doses up to 100 mg per day with complete remission of anxiety. 8 months later, she went to the emergency room for loss of consciousness and tremor of the lower limbs. She was diagnosed with conversive disorder and was prescribed lorazepam up to 3 mg per day. Since then, there has been a worsening in the frequency of syncope occurring up to 10 times a day, limiting her academic and social life. She was evaluated by a cardiologist who diagnosed Pots Syndrome (postural orthostatic tachycardia syndrome) and started treatment with ivabradine and mineralocorticoids. With this treatment, the episodes were drastically reduced and spaced out to 1-2 per week. The dose of lorazepam is decreased until its withdrawal without worsening of the anxious symptomatology.

**Conclusions:**

This disorder consists of an involvement of the autonomic nervous system in which there is a sudden drop in blood pressure together with an abrupt increase in heart rate. Its treatment is based on increasing blood volume with drugs such as corticosteroids as well as postural measures with adequate water intake.

**Disclosure:**

No significant relationships.

